# Improved food recognition using a refined ResNet50 architecture with improved fully connected layers

**DOI:** 10.1016/j.crfs.2025.101005

**Published:** 2025-02-27

**Authors:** Pouya Bohlol, Soleiman Hosseinpour, Mahmoud Soltani Firouz

**Affiliations:** Department of Agricultural Machinery Engineering, Faculty of Agricultural Engineering and Technology, University of Tehran, Karaj, Iran

**Keywords:** Refined ResNet50, Specific connected layers, Optimized hyperparamters, Iranian food, Deep learning

## Abstract

Food consumption has significant effects on human health, particularly in relation to quality control, quantity, freshness, and color. This study focuses on identifying food across 16 categories, divided into breakfast, lunch, and dinner, to evaluate its impact on the human body, specifically in hospital and restaurant settings. The recognition system was used a machine vision system and deep learning algorithms to record food consumption videos, extracting images. After preprocessing, a raw dataset was bulit that consisted of 12,000 images, expanded to 66,000 images through data augmentation. Five deep learning algorithms were used for recognizing food and consumed food. ResNet50 was the best algorithm in comparison to other deep learning architectures. The effect of Hyper-parameters such as data augmentation, batch size, image size, and learning rate on performance of Resnet50 were analyzed. Transfer learning method led us to develop three versions: standard ResNet50, fine-tuned ResNet50, and optimized ResNet50 with a customized fully connected layer. ResNet50 with a specific dense layer was the best development version of ResNet50. This model with Adam optimizer, 10^−3^ initial learning rate, batch size 4, and image size 340 × 640 could recognize various foods with 97.25% accuracy and 0.2 loss. Response time and training time of this architecture compared to other algorithms were confidential; the training process and response time were 5.30 h and 1.2 s. ResNet50 with a specific fully connected layer powerfully could complete tasks with high accuracy and the least time.

## Highlights

• Expanded dataset from 12K to 66K images, boosting model robustness.
• Hyperparameter tuning enhanced model performance and efficiency.
• Analyzed Pre-training and Fine-tuning of ResNet50 architecture.
• Developed ResNet50S with tailored fully connected layers.
• Optimized ResNet50 achieved 5.30h training and 1.2s response time.

## Graphical abstract

**Figure:**
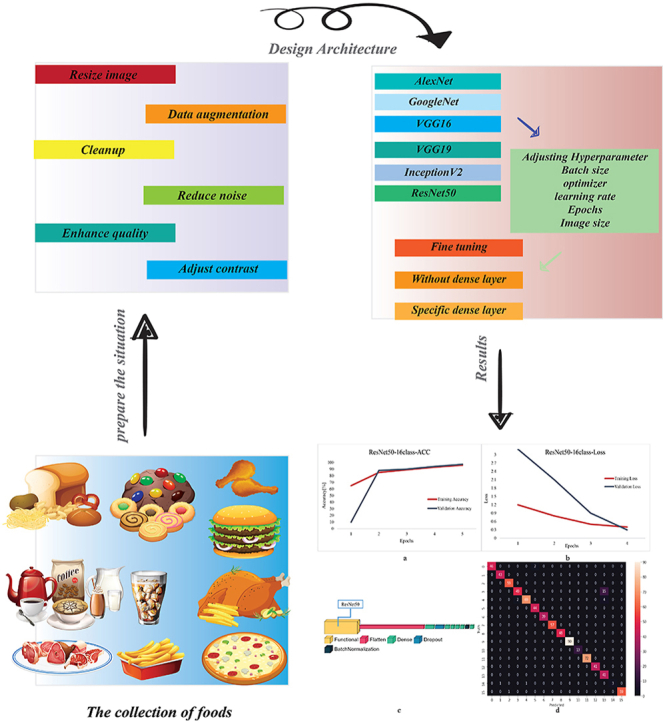

## 1 Introduction

Nutrients like oxygen and water are essential for survival. The human body relies on food for energy, growth, and digestion to absorb nutrients. Food plays a crucial role in reproduction and overall survival, so it's important to consider the selection and types of food consumed. (Council, 2011; Lawson and Glenn, 2021). Quality control is crucial in all industries, especially in grading food products. The food production industry faces challenges in classifying food products for various needs. This includes catering to special dietary needs in hospitals and hotels, managing leftover and consumed food, and analyzing recycled and discarded materials. Additionally, controlling food consumption is essential for individuals with conditions such as overweight, developmental disorders, and diabetes (Day et al., 2021; Levy et al., 2022).

In 2020, diabetes and obesity emerged as significant issues, impacting 2.3 billion children and adults worldwide. Furthermore, many individuals struggle with eating disorders and a lack of nutritional knowledge, affecting around 150 million children. Additionally, foreign tourists often encounter misinformation about the local cuisine of the countries they visit. As a result, developing applications and techniques for identifying food and tracking consumption is essential for societal well-being. (Popkin et al., 2020). Many traditional applications and techniques for analyzing food and monitoring diets are required, such as food frequency questionnaires (FFQs), food quality questionnaires (FQQs), and counting eating operation units (EAUs). These methods classify food and estimate the calories. Accurate and fast recognition of foods is important for controlling obesity, diabetes, or releasing information about products (Despoudi et al., 2021; Wu et al., 2017).

Previous methods have more problems in implementation, such as time of response, accuracy, and not being comfortable. Emerging methods like electronic noses and tongues, image processing, artificial neural networks (ANNs), machine learning (ML), hyperspectral imaging, and MRI imaging are developed for increasing human life satisfaction with accurate recognition, low error rates, and fast and simple implementation in data (Hassan et al., 2025; Mahanti et al., 2024; Ozel and Oztop, 2021). The data set plays a crucial part in the intelligent system and has a great impact on the performance of the model. Malvandi et al. used a portable near-infrared spectrometer and ANN for determining the hardness of dried apples with 95% R^2^ (Malvandi et al., 2022). Liu et al. coupled intelligent algorithms with computer vision for classification of specific tea; deep neural networks could classify flowering stage and tea with 96% accuracy (Liu et al., 2020). Yadav et al. designed an image-capturing system with SVM for recognizing fried potatoes with 98.8% accuracy (Yadav et al., 2018). Raihen et al. classified dried grapefruit with image acquisition, Adaboost, and LightGBM with 98% accuracy (Raihen and Akter, 2024). Cruz et al. aimed to estimate the freshness of eggs by portable near-infrared and SVM with 87% accuracy (Cruz-Tirado, Lucimar da Silva Medeiros and Barbin, 2021).

The integration of computer vision and machine learning in the food industry has transformed classification practices, improving grading systems through precise feature selection like color and texture. While traditional techniques are efficient and adaptable, all of these methods encounter complex and big data; changing the situation of the image acquisition system, type of product, lighting, and angle of the camera has imperfections in accuracy, loss, and time of response (Fan et al., 2020; Finlayson, 2018; Narendra and Hareesha, 2010). Deep learning architectures are especially suited to this role; these architectures extract features automatically, work with complex and big data, respond quickly, and recognize multiple objects. The core concept of deep learning involves employing hierarchical processing with numerous layers of architecture, which are organized in a hierarchical manner. The advancement of deep learning methods has combined two crucial steps in image processing: identifying the most relevant features and categorizing the dataset (Koirala et al., 2019; A. Krizhevsky, I. Sutskever, & G. E. Hinton, 2012).

Deep learning architecture focuses on extracting deep features from images and data, so increasing the complexity and volume of data has a direct impact on the training time and response time of the system. Using deeper architecture requires the best memory and GPU system for computing and converging the speed of the model. Therefore, based on the data set, architectures were selected and implemented. Park et al. used ResNet50, region of interest (ROI), and generative adversarial networks (GAN) for recognizing 7 species of yeast in food with 96% accuracy (Park et al., 2025). Rokhva et al. designed EfficientNetB7 and data augmentation technique for classification 11 type of foods with 96.4% accuracy (Rokhva and Teimourpour, 2025). Din et al. compared 3 models of RiceNet, InceptionV3, and ResNetInceptionV2 for classifying five classes of rice based on quality and accuracy in order: 94%, 84%, and 81.33% (Din et al., 2024). Farooq et al. implemented a convolutional neural network (CNN) for the classification of 7 and 60 categories of food with 94% and 60% accuracy, respectively (Farooq & Sazonov, 2017). All investigations focused on increasing accuracy with pre-trained models such as VGG16, VGG19, InceptionV3, EfficientNetB7, ResNet101, and 152.

In this investigation, the dataset consists of a variety of features, such as a large dataset, multiple objects, and specific foods across various conditions. On one hand, the research involves high complexity and massive datasets, while on the other hand, it aims to reduce response time, training time, and computational demands while preserving accuracy and minimizing algorithmic loss. The use of deeper algorithms increased many parameters; therefore, the primary objective was to identify lightweight algorithms suitable for developing solutions based on this complex dataset.

The remainder of this paper is structured as follows: Section two describes the dataset construction, the data collection process, and the configuration of the imaging system. Section three gives an overview of convolutional neural networks and details the system architecture for food consumption recognition. Section four presents the results obtained from the deep learning approach, with a brief discussion provided in Section five. Finally, Section six concludes the paper.

## 2 Data set description

### 2.1 Data acquisition

**Fig. 1:**
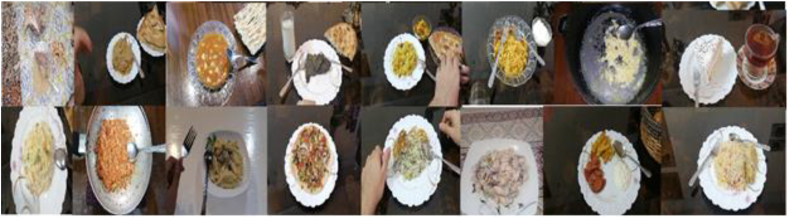
Collection images of data.

Collecting samples of food products is crucial for creating an effective and efficient management system. The production and consumption of food have direct impact on human life and the environment. Evaluating and controlling the production and consumption of food products, can reduce waste, preserve natural resources, maintain a clean environment, and manage energy effectively. In various countries, food products exhibit a wide range of diversity based on their unique cultures and civilizations. This study focused on identifying the variety of Iranian food products by selecting a number of them for sampling. A total of 16 classes of food products were chosen for imaging during the sampling phase. Sampling of food products from consumption start to finish was documented in a video. Example images of selected food products are shown in Fig. 1.

**Fig. 2:**
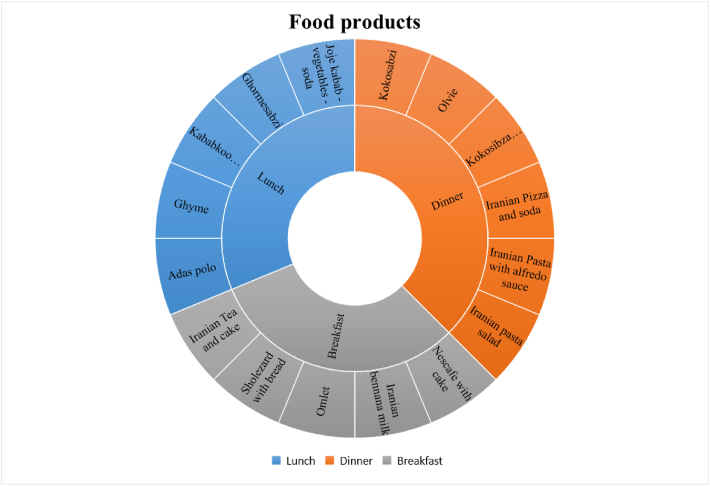
Statistics meals, inner circle shows 3 main meal categories. The other circle shows the details of the main categories of the food.

Food products were divided into three main meals: breakfast, lunch, and dinner. In each meal, there were several types of food classes, and it was tried to use one main meal in each class along with a drink or appetizer during imaging. Food products with three main meals and 16 classes of selected subsets, with the names of the food products shown in Fig. 2.

**Fig. 3:**
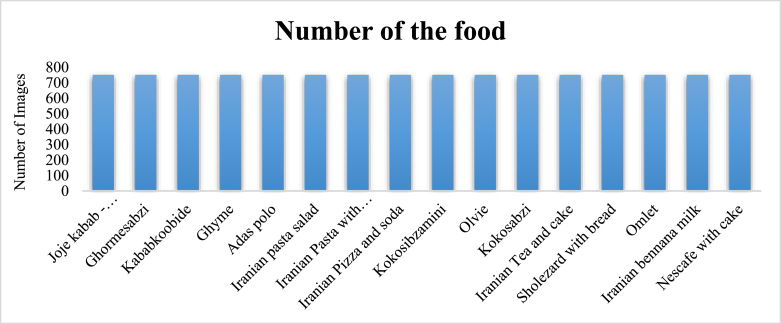
The number of images from each category.

The system for identifying consumed food products was initially implemented by taking images of the food products at the time of consumption. However, for improved analysis and ease of use, various cameras were installed to capture videos of each product during consumption. Initially, 16 classes of food products were selected for creating the data set. These videos were processed using the Python programming language, where images were extracted at 30-s intervals. The extracted images were then transferred to preprocessing datasets and deep learning systems for further analysis. The data set consisted of 12,000 images that are shown in Fig. 3.

### 2.2 Imaging system

**Fig. 4:**
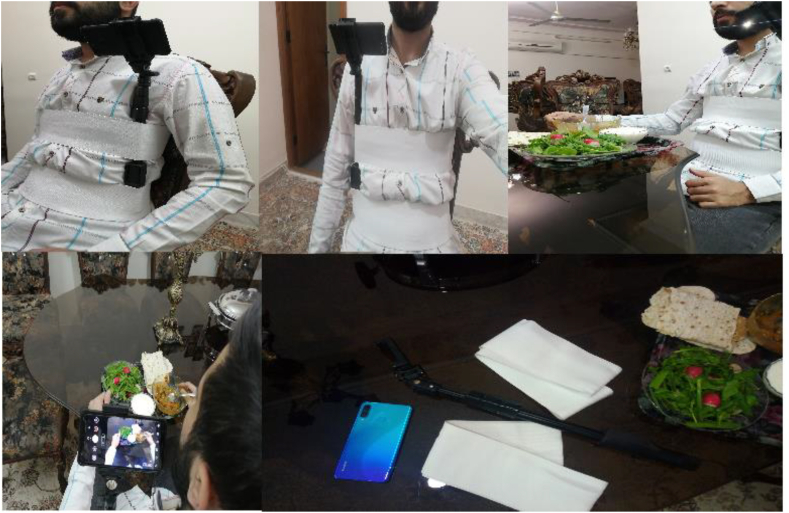
The placement of cameras and equipment for imaging food products.

The imaging system for identifying consumed food items comprised a camera affixed to a monopod, secured to the user with straps. A mobile phone with a camera was mounted on the monopod, positioned on the user's left side, with the camera aligned nearly parallel to the shoulder at an approximate angle of 160°. The angle varied dynamically as the user moved during food consumption, affecting each video frame. The setup was adaptable for diverse conditions, incorporating various lighting sources, cameras, and environments, including moonlight, LED lamps, and devices such as the Canon and Nikon cameras, Samsung A73, Huawei P30 Lite, and Xiaomi Poco F5. Video data were transferred to a laptop for processing, ensuring efficient handling of large files. The laptops, equipped with AMD Ryzen 5 3500U CPUs, AMD Radeon Vega 8 GPUs, 8 GB RAM, and 128 GB SSDs, supported image analysis and deep learning tasks. For computationally intensive processes like training deep learning models, Google Cloud services were employed. Specifically, Google Colab facilitated the execution of algorithms with its GPU and TPU support, providing the computational power necessary to process the substantial data generated and refine models effectively, position all equipment is shown in Fig. 4.

#### 2.2.1 Imaging of consumed food products

**Fig. 5:**
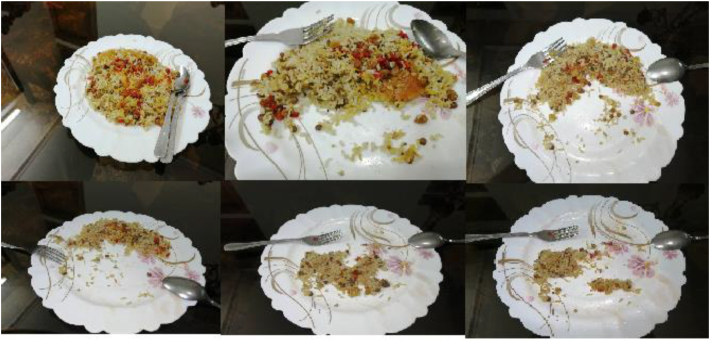
Pictures of the desired product from the beginning of use to the end of its use.

**Fig. 6:**
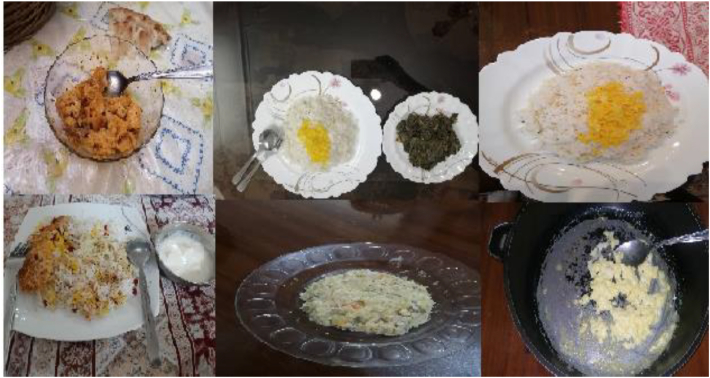
Consider various locations for product photography.

The images show the stages of consuming a food product, extracted from videos using machine vision and Python programming, Fig. 5. Shows the stages of food consumption. The videos were taken in different conditions. There are six examples of food in multi situation for recording video during the food consumption that shown in Fig. 6.

## 3 Deep learning method (convolutional neural networks)

The convolutional neural network is a deep learning architecture that has proven to be highly effective in improving performance, accuracy, sensitivity, and machine vision (Hubel and Wiesel, 1962). convolutional neural network architecture, introduced in 1962, drew inspiration from the structure of a cat's eye. It was the first of its kind and was initially used for classifying handwritten figures (Hecht-Nielsen, 1992). However, CNNs gained significant popularity after 2012 due to advances in GPU computing. It wasn't until 1999, when the speed and performance of computers increased along with the availability of graphics cards, that image net datasets were created, paving the way for a new era in deep learning. In 2012, Krizhevsky et al. achieved success in the ILSVRC competition with their deep learning model called AlexNet, sparking growing interest and leading to the design of various models with different functions (LeCun et al., 1989) (A. Krizhevsky, I. Sutskever, & G. Hinton, 2012).

**Fig. 7:**
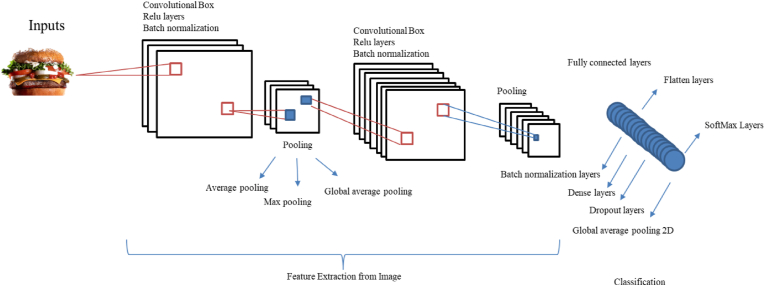
The structure of CNN used for feature extraction and recognition of the specific objects.

Convolutional Neural Networks (CNN) are a prominent deep learning technique that involves the training of multiple robust layers. Typically, CNNs consist of three main types of layers: convolutional layers, pooling layers, and fully connected layers. Each type of layer performs a distinct function. Architecture of convolutional neural networks was shown in Fig. 7, which is designed for image classification through a layer-by-layer process (Sajedian et al., 2019). Table 1 reported the information of 6 popular deep learning architectures based on parameters and accuracy.

**Fig. 8:**
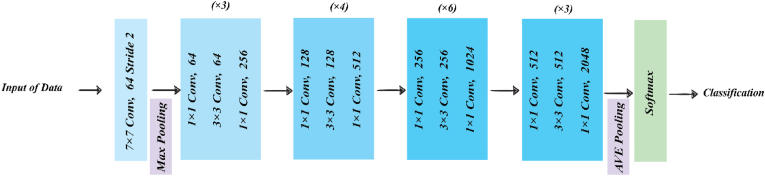
ResNet architecture.

**Tabel 1:** Information of 6 popular deep learning architectures.

Model	Year	Total Layers	Parameters [Million]	Top-1 Accuracy	Top-5 Accuracy	References
AlexNet	2012	8	60	63.3%	84.6%	(Krizhevsky et al., 2012a, Krizhevsky et al., 2012b)
GoogleNet	2014	22	6.8	69.8%	89.6%	Szegedy et al. (2015)
VGG16	2014	16	13.8	71.5%	89.8%	Simonyan (2014)
VGG19	2014	19	14.3	71.3%	89.9%	Simonyan (2014)
InceptionV2	2015	42	11.2	74.7%	92.2%	Szegedy et al. (2016)
ResNet50	2015	50	25.6	76%	93.0%	He et al. (2016)

### 3.1 ResNet architecture

ResNet (Residual Networks) architecture was introduced to address the issue of gradient fading in deep network by He et al. (2016). This unique architecture includes skip connections, allowing the model to bypass layers and transfer features directly to higher layers. ResNet comes in different versions, such as ResNet50 and ResNet101, with 50 and 101 layers, respectively. In the 2015 ILSVRC competition, this architecture achieved first place with an error rate of 3.57%. Due to its high depth and accuracy, it is widely used in various computer vision tasks. Fig. 8 provides details of this architecture.

### 3.2 ResNet50 with specific dense layer

The food dataset that was created was very extensive and required a high-performance and accurate architecture. After analyzing various deep learning architectures based on factors such as accuracy, loss, response time, learning rate, batch size, image size, and total parameters, ResNet50 was chosen. The dataset consisted of 16 classes, each with numerous variations, making it challenging to identify the type of food. Additionally, the dataset was collected during consumption, requiring the model to recognize incomplete or partially visible food items. To achieve high accuracy, low loss, and minimal response time with fewer parameters and runtime, a specific architecture was designed. Firstly, the dataset needed to be expanded using data augmentation techniques, which greatly impact accuracy and loss. The batch size and image size were determined based on the model and accuracy results. Afterwards, the optimizer was chosen, and the learning rate was set for deep learning algorithms. Finally, the specific dense layer of ResNet50 was designed. The significance and settings of these fully connected layers in deep learning will be discussed in the subsequent sections.

#### 3.2.1 Data augmentation

**Fig. 9:**
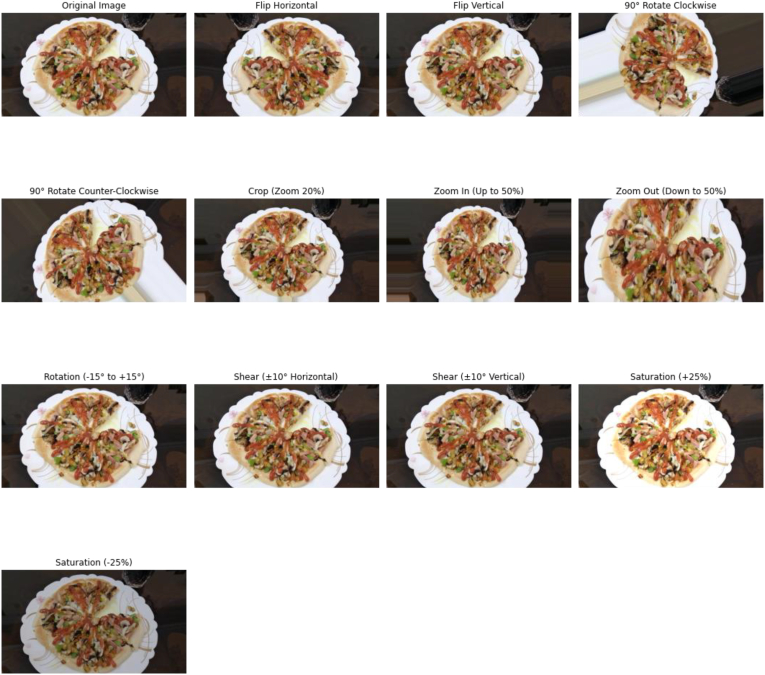
The process of the data augmentation technique.

**Fig. 10:**
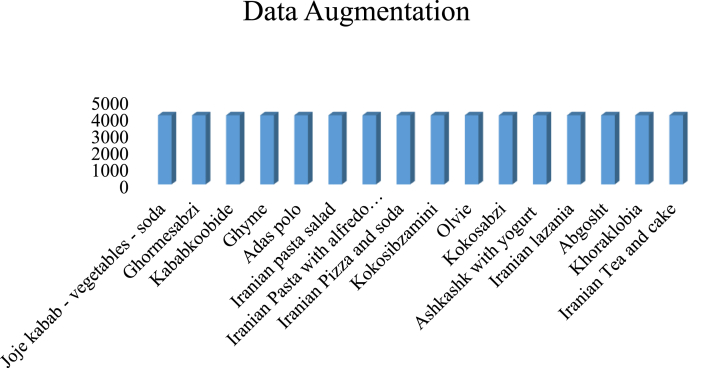
Number of images after implementing the data augmentation technique.

Data augmentation is a technique for enhancing data by changing the size, intensity, color, and lighting for deep learning algorithms. This technique by expanding the diversity of a training dataset to original data sets was especially useful for improving the efficiency of deep learning algorithms, preventing over-fitting, and increasing the generalization ability of models (Taylor and Nitschke, 2018). Some data augmentation that was used in this investigation was presented in Fig. 9. First, rotate the image with a specific angle of about 10 and 15° to induce the model to be able to recognize the multiple objects. Second, translating the food to left and right, the model could resist changing the condition of the object. Third, shearing the image made a new image with a new angle of view. Fourth, zooming in or out the image was the way to train the model for determining the object on different scales. Fifth, increasing or decreasing the contrast and intensity of the image with brightness adjustment was a route for a deep learning algorithm that worked in different lightning conditions. Five steps of data augmentation were done, and data was increased from 12000 to 66000. The number of image and data augmentation techniques is shown in Fig. 9, Fig. 10.

#### 3.2.2 Batch size

**Fig. 11:**
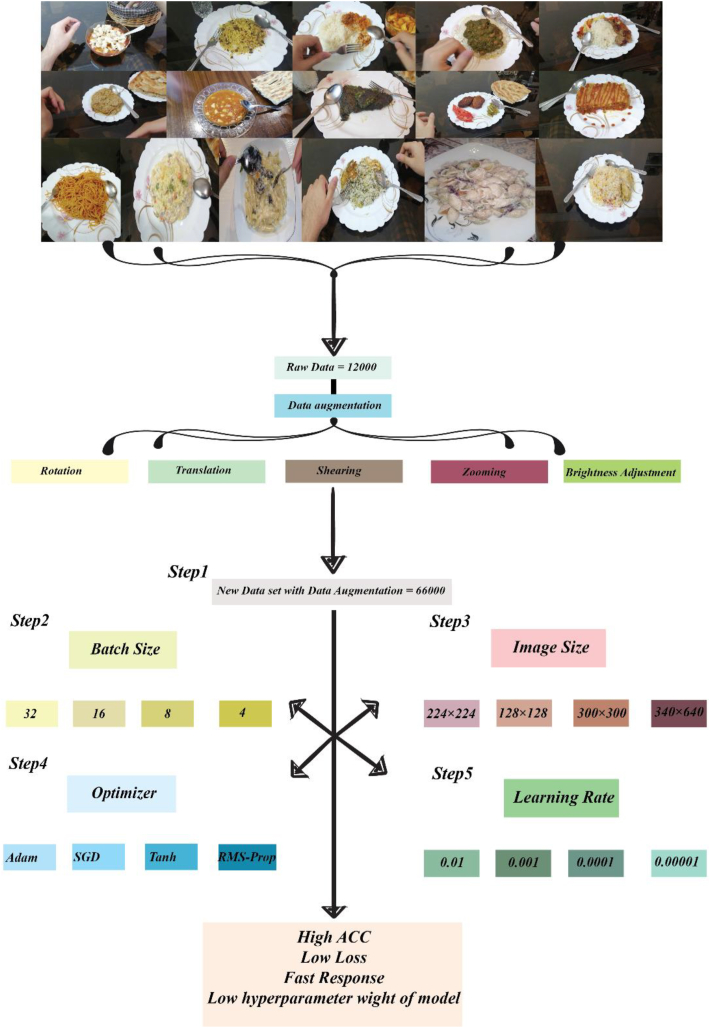
Workflow of preprocessing in deep learning for higher accuracy and least loss. Step 1, data augmentation. step 2, number of batch sizes. step 3, size of image for model, step 4, optimizers, and step 5, determining the level of learning rate.

Batch size was indicated as the number of images that at a time were given to the generator or model for processing, loading, and predicting. Batch size controls how many images are loaded; this technique could help to manage the big data that couldn't load at a time and decrease the usage of the GPU and CPU. In general, batch size could control memory usage and training time; these factors depended on the amount of batch size and size of the image (Aldin and Aldin, 2022; Yong et al., 2020). In this paper, batch size was selected based on accuracy and loss that show the best result at least time. Fig. 11, step 2, shows the numbers of batch sizes that were used.

#### 3.2.3 Image size

The size of the images is the crucial parameter in preprocessing data for the deep learning algorithms; this parameter has a great impact on the training efficiency, model performance, hardware constraints, and architecture requirements (Shorten and Khoshgoftaar, 2019). This study employed numerous algorithms, initially determining the image size according to the model's requirements. After showing results, the size of images was changed because images captured during the consumption and the image that was extracted from the frame of video had many situations, like volume of food, illustration, angle, and changing the situation of other objects in the picture. Fig. 11 step 3 illustrates the size of the image; the dimension of the image was devised in 340 × 640 for more accuracy, low loss, and hardware constraints.

#### 3.2.4 Optimizer

Optimizer is a method that is used to adjust the weight of algorithms for minimizing the loss function and increasing the accuracy, and overall, this function optimizes the deep learning algorithms with preventing overfitting, enhancing the ability of the model to predict unseen data, and improving coverage speeding (Desai, 2020; Sun, 2020). Many optimizers were used in this investigation, as shown in Fig. 11 step 4, but Adam was the best optimizer in terms of predicting objects with high accuracy and lasted loss.

#### 3.2.5 Learning rate

The learning rate is an important hyperparameter in the training process of deep neural networks. The amount of learning rate has a direct impact on underfitting and overfitting; the size of the weight update is shown in Fig. 11 step 5. Thus, finding the right learning rate for deep learning architecture is essential for maximizing its accuracy and efficiency during training. Techniques like learning rate schedules, cyclical learning rates, or using optimizers like Adam can help dynamically adjust the learning rate to improve performance. Adam had best performance comparison other optimizers.

#### 3.2.6 Specific fully connected layer

**Fig. 12:**
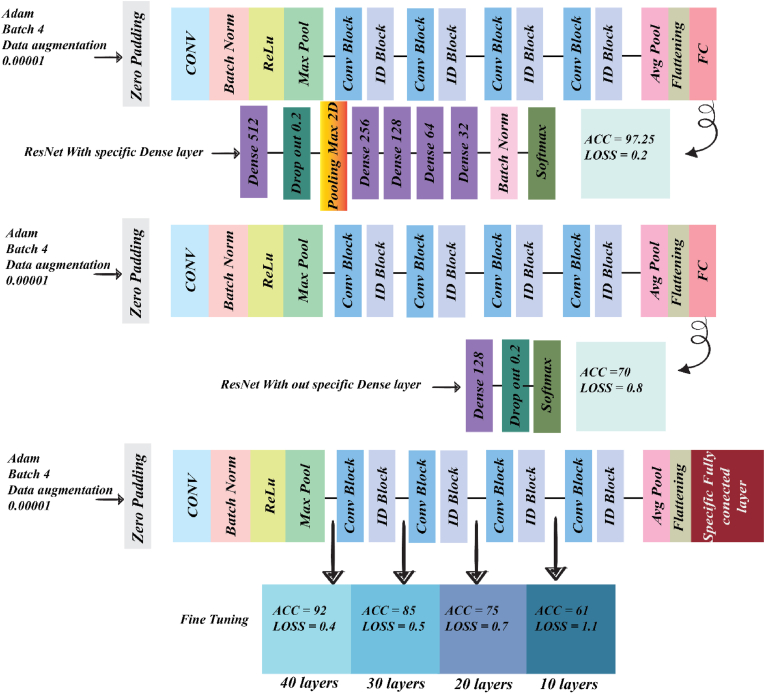
Structure of deep learning: this graph illustrated the ResNet50 in three different configurations. First, ResNet50 with a specific dense layer was shown. Second, fine-tuning was done at 4 steps, and the last step was ResNet50 without fixing the dense layer. The performance of ResNet50 compared with and without specific dense layers highlights the effects of fine-tuning at different stages.

A fully connected layer (FC) has an important task in classification and analyzing the features of images. Every neuron was connected to every neuron in the previous and next layer; this layer worked like a multi-layer perceptron with big differences. First, feature extraction was done by pre-trained algorithms before FC. After that, many layers like dropout, batch normalization, pooling, and dense with different activations could be used (Basha et al., 2020). So, after 5 step workflows of preprocessing, adjustment of the fully connected layer was an important role. Amount of each parameter in fully connected, value and condition of dense layer, dropout, batch normalization, and existence and lack of another layer were crucial. ResNet50 with a specific fully connected layer was achieved after many tests; Fig. 12 step 1, shows this architecture.

#### 3.2.7 Fine-tuning ResNet50

The process of fine-tuning involves transferring knowledge from related domains and adjusting certain network parameters, such as convolution layers, to better suit specific tasks. This can lead to improved performance using less data and computational resources (Cetinic et al., 2018). In this study, we fine-tuned ResNet50 in four steps, and at each step, the model was validated. Fig. 12 shows the results of the fine-tuning process and indicates the layers where fine-tuning was performed.

## 4 Results

### 4.1 Performance metrics

Confusion matrix plays a significant role in evaluating the performance of the model. A confusion matrix in binary classification is made up of four metrics. True positive (TP), false positive (FP), true negative (TN), and false negative (FN), which are used to evaluate the prediction of the convolutional neural network that is shown in Table 2. Overall, there are many ways for measuring the performance of deep learning algorithms. They include precision, recall, accuracy, and f1-score.

(1) $$accuracy=\frac{TP+TN}{TP+TN+FP+FN}$$

(2) $$precision=\frac{TP}{TP+FP}$$

(3) $$Recall=\frac{TP}{TP+FN}$$

(4) $$F1=\frac{2\;\mathrm{PR}}{TP+1/2\left(FN+FP\right)}$$

**Table 2:** Confusion matrix.

	Predicted results		
Real situation		1	0
0		**TP**	**FN**
1		**FP**	**TN**

### 4.2 Workflow of identifying food and consumed food

**Fig. 13:**
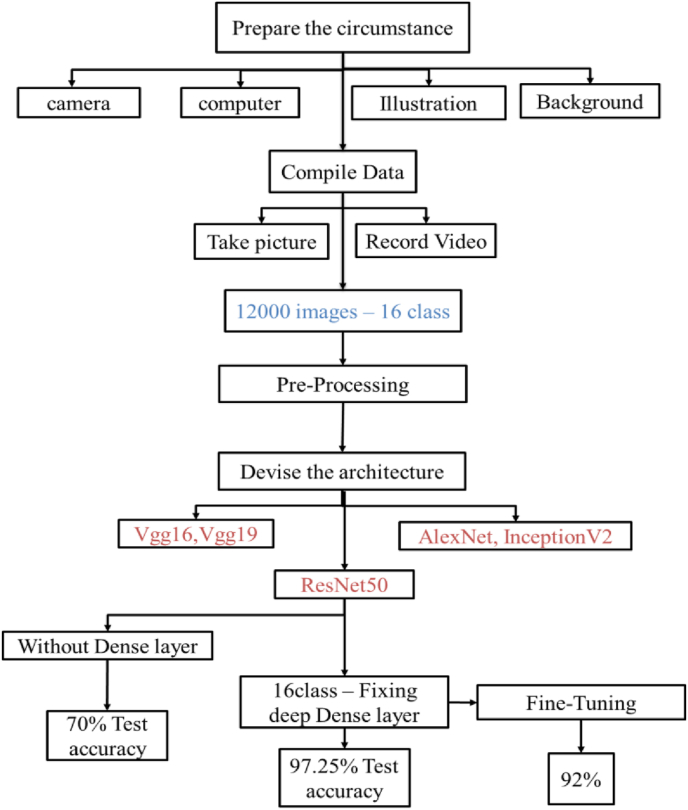
Illustrates the circumstances of the work. The situation was adjusted to collect data sets and use a deep learning architecture to achieve high accuracy.

Identifying consumed food products can be a challenging task due to incomplete information about the products. Additionally, hardware and data limitations contribute to poor network performance in this sector. To address this, we have trained a network using 12,000 images for 16 classes of food products without data augmentation. The results demonstrate that ResNet50 and settings of fully connected layers were highly effective. Several well-known deep learning architectures were employed in this study, including AlexNet, GoogleNet, VGG16, VGG19, InceptionV2, and ResNet50. Initial experiments with VGG16, VGG19, AlexNet, GoogleNet, and Inceptionv2 did not yield satisfactory results. In contrast, ResNet50, which has been successfully utilized in numerous studies to optimize dense layers, demonstrated better potential for our application. Recognizing consumed foods and foods poses significant challenges due to the incomplete nature of the food items, coupled with hardware and data limitations that can adversely affect network performance. The workflow of the process and the associated results were illustrated in Fig. 13.

### 4.3 Comparison performance of different models and preprocessing methods in the process of training

Different deep learning architectures were used to identify consumed food products. An example of the architectures used in this study to obtain higher accuracy is shown in Table 3. Several parameters affect deep learning architectures, including image image-sizes, the number of images, settings of fully connected layers, the number of epochs, and hyperparameters. First, the image-size of the images was the normal input size that was defined in the sitting of the model, and the number of epochs was determined based on overfitting and underfitting rates. Size of the images and batch size were the important parameters in modeling. The image-size of the image had a direct impact on accuracy and loss; increasing the image-size of the image from 224 × 224 to 340 × 640 was achieved after many tests that many runs weren't reported. The amount of the batch size was determined by the number of input images in the deep learning method; for big data, batch size is very crucial. Increase or decrease the batch size has a great impact on the overfitting and underfitting.

**Table 3:** The performance of the architectures with best hyper-parameter developed for consumed food recognition.

Architecture	Epochs	Image-Size	Train accuracy	Test accuracy	classes	Optimizer	Batch size
AlexNet	25	224 × 224	19%	20%	16	Adam	16
GoogleNet	25	224 × 224	20%	22.5%	16	Adam	16
VGG 16	25	224 × 224	23.5%	25%	16	Adam	16
VGG 19	25	224 × 224	24%	27.87%	16	Adam	16
InceptionV2	25	299 × 299	29.5%	31.5%	16	Adam	16
ResNet50	25	224 × 224	45%	46.5%	16	Adam	16
ResNet50	20	400 × 400	49%	51%	16	Adam	16
ResNet50	20	340 × 640	56%	58%	16	Adam	16
ResNet50	20	340 × 640	59%	60%	16	Adam	8
ResNet50	20	340 × 640	63%	65%	16	Adam	4
ResNet50 with Data augmentation	20	340 × 640	69%	70%	16	Adam	4

**Table 4:** The performance of ResNet-50S with different sizes of trainable parameters.

Architectures	Epochs	Accuracy	Precision	Recall	F1-Score	MAE Test	MSE Test	RMSE Test	Class and optimizer
ResNet-50S-Fine tuning 10 layer	5	61%	60%	60%	61%	0.3	0.62	0.70	16-Adam
ResNet-50S-Fine tuning 20 layer	5	75%	76%	75.5%	76%	0.25	0.75	0.80	16-Adam
ResNet-50S-Fine tuning 30 layer	5	85%	85%	86%	84%	0.1	0.88	0.85	16-Adam
ResNet-50S-Fine tuning 40 layer	5	92%	93%	93%	92.5%	0.05	0.90	0.89	16-Adam
**ResNet-50S**	**5**	**97.25%**	**96.3%**	**97%**	**97%**	**0.03375**	**0.991**	**0.95**	**16-Adam**

**Fig. 14:**
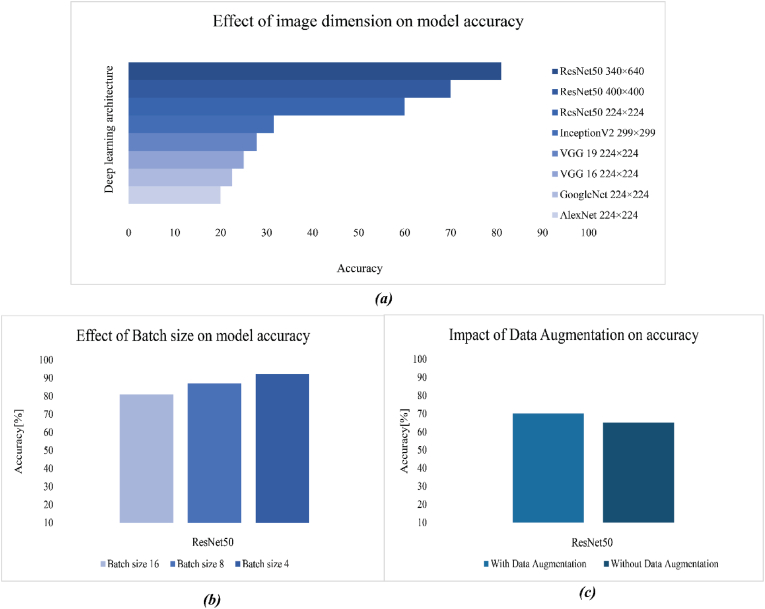
Effect of image image-size, batch size, and data augmentation technique on deep learning architecture.

The performance of the deep learning architecture that is used for classifying food and consumed food is shown in Fig. 14a. Deep learning architecture with the actual size of image that sitting architecture introduced was used, but the accuracy was not good. Images of foods were captured during the consumption with a multicamera, situation, and different size of food, so images with low resolution were not enough for the model to recognize the type of the foods. After test 6 deep learning architecture, we decided to increase the image-size of the image. Increasing the image-size of the image had a direct impact on accuracy; Fig. 14a illustrates that ResNet50 with a 400 × 400 image image-size showed 51% accuracy, and ResNet50 with a 340 × 640 showed 58% accuracy. After that, for higher accuracy, data augmentation was done, and batch size decreased from 16 to 8 and next to 4. Data augmentation and decreasing the batch size showed the positive results, and accuracy increased in Fig. 14b and c.

### 4.4 Effects of hyper-parameters on model performance

#### 4.4.1 Optimization

**Fig. 15:**
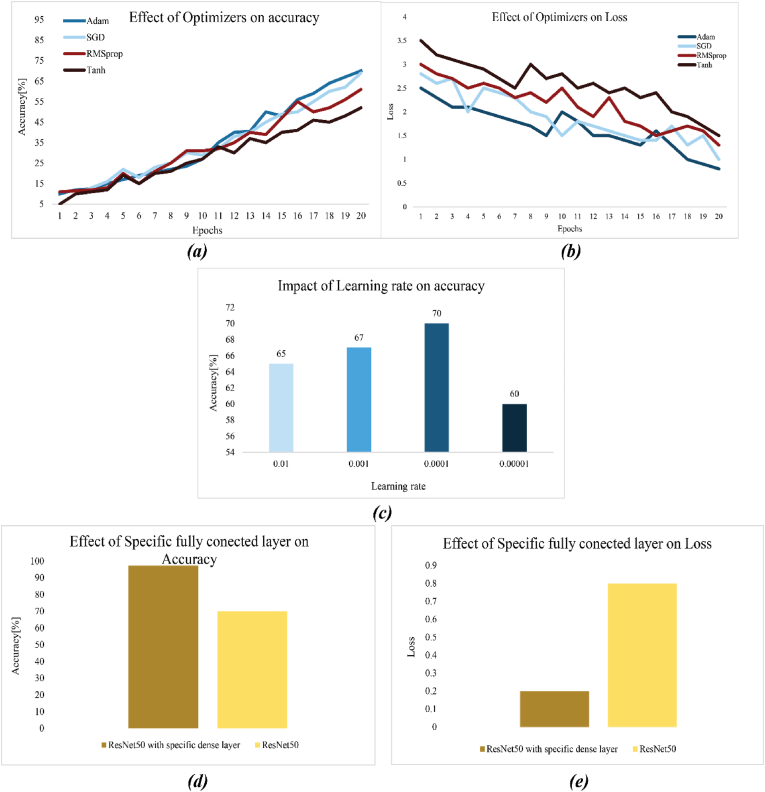
illustrates the impact of the optimizer, learning rate, and specific fully connected layer on the deep learning model. (a, b) shows the different optimizers and effects of them on accuracy and loss of ResNet50. (c), increasing the learning rate showed positive results. (d, e), demonstrated the impact of a specific fully connected layer on accuracy and loss.

The hyper-parameters of the model have a certain influence on the model performance, and suitable hyper-parameters can improve the convergence speed and accuracy of the model. The learning rate is an important factor affecting the convergence speed of the model. In this study, four different learning rate optimization methods were adopted in model training, as presented in Fig. 15a and b. The model without a specific dense layer test with four learning rate optimizations, Tanh, and RMSprop after 20 epochs showed the same performance in accuracy and loss. RMSprop and Tanh had 61%, 52% accuracy, and 1.3%, 1.5% loss in the ResNet50 model for classification food and consumed food. These results were not satisfied for the model, so the optimizer of the model changed to SGD and Adam; the accuracy and loss of the model had better performance after analysis and testing the model. Adam and SGD had 70%, 69% accuracy, and 0.8%, 1% loss in classification 16 of food. Adam was the better learning rate optimization, and the same phenomenon was found in the research of Feng et al. (2019). Consequently, Adam was chosen as the learning rate optimization method.

#### 4.4.2 Initial learning rate

Adam optimization algorithms are not highly sensitive to the initial learning rate, but the initial learning rate is the important hyperparameter that can generally impact the convergence speed of the model for the classification of 16 classes of food and consumed food. A convenient initial learning rate can speed up the convergence of the model. Four different initial learning rates (10^−2^-10^−5^) were used in the process of model training. The result of each initial learning rate on ResNet50 without a specific dense layer is shown in Fig. 15c. When the initial parameter was increased from 10^−2^ to 10^−4^, the accuracy of the model was increased, but after that, accuracy decreased because 10^−5^ wasn't close to the optimal value of the learning rate. Initial learning rate of 10^−4^ could quickly find the optimal gradient descending direction. According to the accuracy and convergence speed of the model, the initial learning rate of 10^−4^ was chosen to train the model.

#### 4.4.3 Specific fully connected layer

A fully connected layer is the important section of deep learning architecture. Pre-train models have specific tasks like feature extraction. After the analysis of the images with the pre-train model, features were extracted and all the information transferred to the flattening or global average pooling 2D. After that, all features were collected and sent to the dense layer of the fully connected layer. Fully connected layers are essential for integrating learned features, reducing image-sizeality, and providing final predictions, making them a critical part of most neural network architectures. Overall, the fully connected layer was the important part of our model; models without this part show 70% accuracy and 0.8% loss, but when sitting of the fully connected layer after many tests were adjusted, high accuracy and low loss appear. Sitting, type of layer, and layer placement were very crucial. Our specific, fully connected layer was constructed from five dense layers, one dropout, one batch normalization, and pooling max. Placement and amount of each layer were very important; the dense layer started at 512 and finished at 32; between the dense layer used dropout, pooling max, and batch normalization. All the parameters and placement of the layer were achieved after many tests. The result of the model with a specific fully connected layer was different from the model without this layer, as illustrated in Fig. 15d and c. The accuracy and loss of ResNet50 with specific fully connected Adam learning rate optimization, 10^−4^ initial learning rate, and batch size 4 were 97.27% and 0.2%.

### 4.5 Comparison model with fine-tuning

**Fig. 16:**
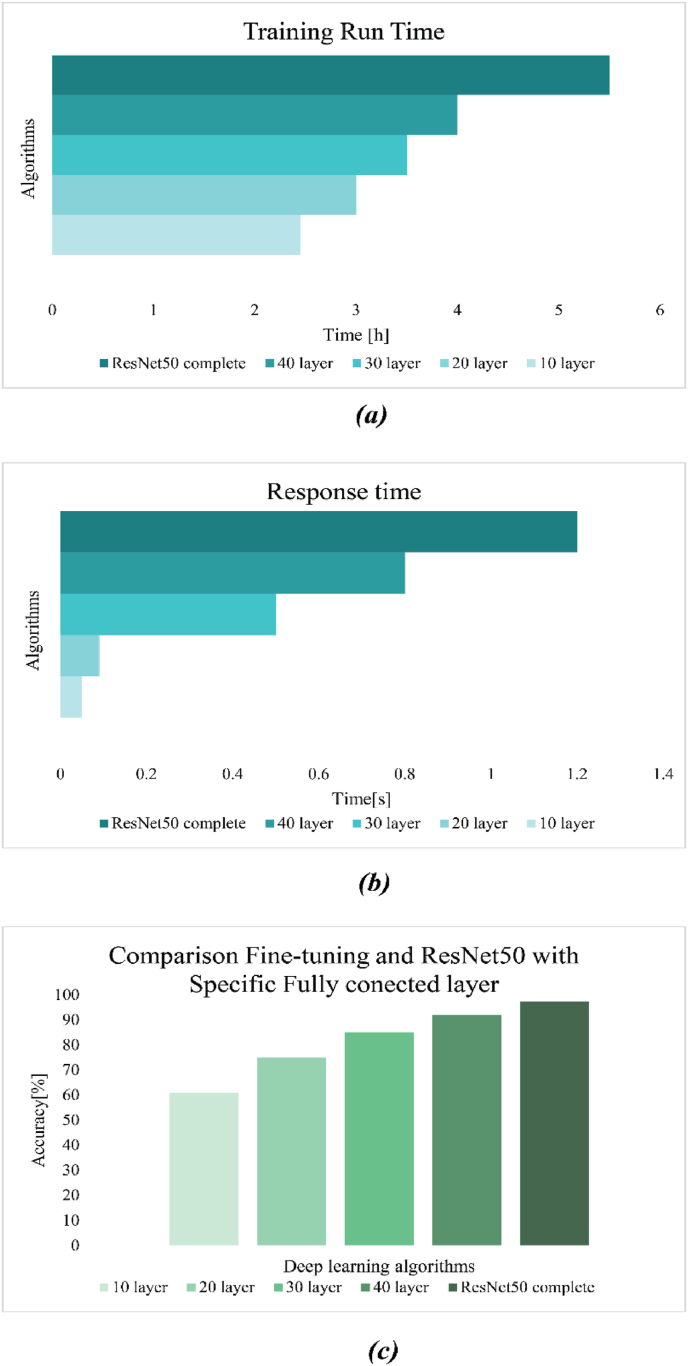
Demonstrated the run time, response time, and comparison performance of fine-tuning models with ResNet50. (a), show the training time of ResNet-50 with 4 different fine-tunes and ResNet-50 without fine-tuning. (b), illustrated the response time of the models, and (c) comparisons model performance in classification the object.

To explore the effects of different fine-tuned layers in ResNet-50, comparison experiments of four different fine-tune layers (10, 20, 30, 40) were conducted. Fine tuning was implemented on ResNet50 with a specific fully connected layer, and the results of these models are shown in Fig. 15. Fine tuning of the initial layer did not show good accuracy; the 10th and 20th convolutional layers of ResNet50 primarily capture low-level visual features such as edges, textures, shapes, and colors. In contrast, the 30th and 40th layers extract high-level semantic features very significantly across different datasets. It is expected that fine-tuning of the lower layers in ResNet-50 has little improvement in performance since the model already extracts most general low-level features of an image. Therefore, beyond the 20th layer, changes to the layers do not substantially improve the performance. That simply means its generalization capability is not considerably affected in case fine-tuning involves more than 20 layers. Also, beyond layer number 40, the performance on fine-tuning stays unchanged; hence, the higher semantic feature representations learned by ResNet-50 are strong and suitable for the given task. Thus, more fine-tuning does not bring significant improvement in the predictive power of the model. However, the high-level semantic features of the model were closely related to the specific dataset. Therefore, the performance was significantly improved by fine-tuning 30 and 40 layers of ResNet-50. Finally, the ResNet-50 was fine-tuned with 40 layers. The results of fine tuning include response time, training time, and accuracy, as shown in Fig. 16a, b and c. Training time and response time of model depended on value and amount of trainable hyperparameter of model. When the model was deeper and total trainable hyperparameter increased in contrast, response time and training time increased. ResNet50 with the 10th layer has the least training time and response time, but ResNet50 with the complete trainable parameter has the highest response time and training time but has a higher accuracy of about 97.25%, as shown in Fig. 16c.

### 4.6 Design ResNet-50S

**Fig. 17:**
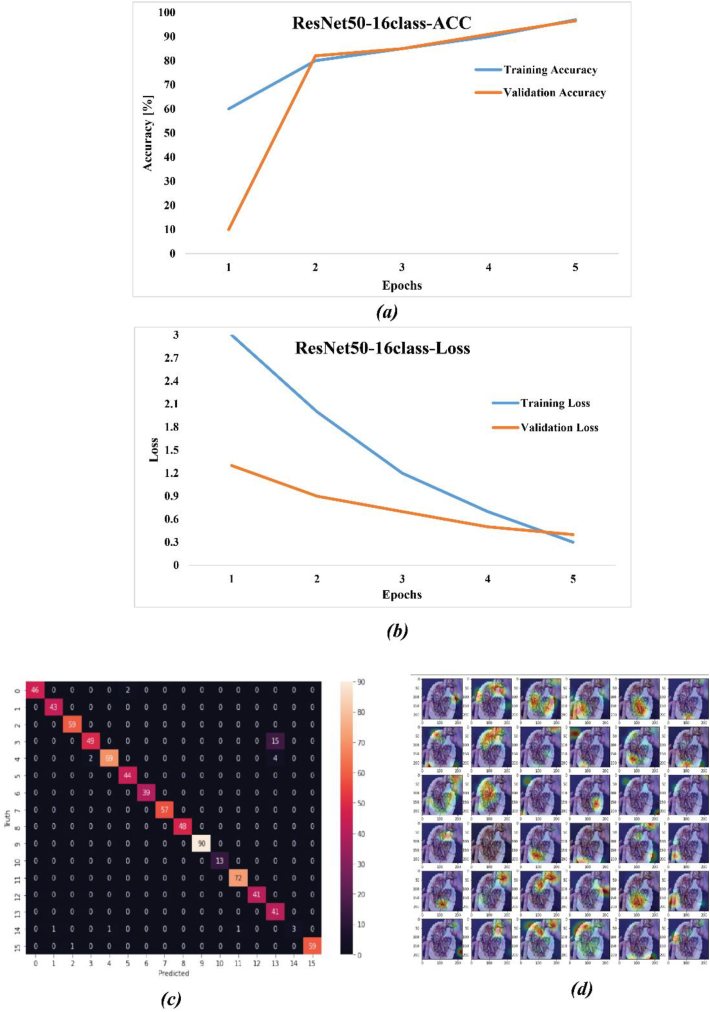
Illustrated result of ResNet50 with a specific fully connected layer. (a) demonstrate the training and validation accuracy. (b) show a graph of the training and validation losses, and (c) illustrate the confusion matrix. (d) Heat map for multiple layers of the ResNet50 network without a specific dense layer.

**Fig. 18:**
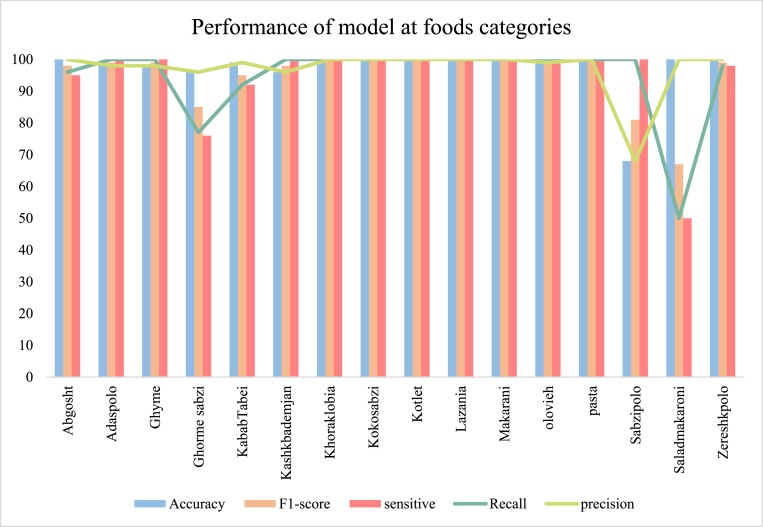
The graph demonstrated the performance of ResNet-50S in classifying each class with accuracy, F1-score, sensitivity, recall, and precision.

The ResNet-50S was a modified version of ResNet-50 with a specific fully connected layer and performance of model with different fine-tuning is shown in Table 4. A comparative analysis was conducted to evaluate the performance of fine-tuned ResNet-50 models at different depths. The assessment was based on key metrics including mean absolute error (MAE), mean squared error (MSE), root mean squared error (RMSE), and test accuracy. The models were fine-tuned at 10, 20, 30, and 40 layers, in addition to a non-fine-tuned ResNet-50, all trained for 5 epochs using the Adam optimizer with an initial learning rate of 10^−4^ and a batch size of 4. The results highlighted the impact of fine-tuning on predictive accuracy and error reduction in the models. Notably, the non-fine-tuned ResNet-50S exhibited the highest accuracy and least loss. Fig. 17a and b illustrate the training and validation process, while Fig. 17a depicts the model's accuracy of correctly identifying foods and consumed foods with 97.25% accuracy. The error reduction during the training process is demonstrated in Fig. 17b, with the error dropping to less than 0.2% after 5 epochs. The confusion matrix for 16 classes is presented in Fig. 17c. The Grad-CAM (Gradient-weighted Class Activation Mapping) visualizations of food images processed by a ResNet50 model without optimized dense layers were illustrated in Fig. 16c. The results indicate that, without an adjusted fully connected layer, the ResNet50 model could not accurately localize and identify the distinctive characteristics of the food items, and this Grad-CAM in principle shows the importance of the fully connected layer to recognizing the object. provides a comprehensive summary of various statistical indicators used to evaluate the performance of a ResNet-50S in classifying different types of foods in Fig. 18. This figure presented Accuracy, F1-score, Sensitivity, Recall, and Precision offer a multifaceted evaluation of the model's efficacy and reliability across multiple food categories. The ResNet-50S could perfectly identify food and consumed food with high performance that showed in Fig. 18, but in some classes, like Sabzipolo, Saladmakarni, and Ghormesabzi, it didn't show high accuracy, precision, and recall because this class had many similarities to other classes and recognizing consumed foods was very difficult. Overall, ResNet-50S was the best model that could identify the food and consumed food with 97.25% accuracy and 0.2% loss.

## 5 Discussion

Food and consumed food are the crucial part of human life. Recognizing the food and consumed food have great impact on life style, environment and recycle management. There has been introducing many recognitions system based on e-nose, image processing and experimental analysis. Computer vision is the traditional method of image processing and deep learning is the new way. Kozłowski et al. (2019), investigated on recognition the quality of beer in 9 classes. They could design convolutional neural network and transfer learning for recognizing the beer in 9 class of quality with 93% accuracy. Huang et al. (2022), design the deep learning architecture for classification soybean seed. They design soybean network (SNet) based on deep learning algorithms with 1.2 million parameters for classification soybean in 6 classes. SNet architecture could classify soybean with 96.2% accuracy. Kaushal et al. (2024) investigated on computer vision and deep learning-based approaches for detection of food nutrients/nutrition: New insights and advances. Computer vision-based approaches have emerged as promising alternatives that enable rapid and non-destructive analysis of various nutritional parameters in foods. They summarized computer vision applications in meat processing, grains, fruits and vegetables, and seafood. They reviewed recent advancements in computer vision and deep learning-based algorithms employed for food recognition and nutrient estimation. They announced deep learning algorithms was the best nondestructive algorithms in food recognition and classification. Bishop et al. (2021), work on Automatic classification of takeaway food outlet cuisine type using machine (deep) learning for identifying and analysis the obesity and impact on diet. They used machine (deep) learning, and specifically a Long Short Term Memory variant of a Recurrent Neural Network, to develop a predictive model trained on labelled outlets (n = 14,145), from an online takeaway food ordering platform. They validated the accuracy of predictions on unseen labelled outlets (n = 4000) from the same source. Although accuracy of prediction varied by cuisine type, overall the model (or ‘classifier’) made a correct prediction approximately three out of four times and deep learning algorithms was the confidential method for recognition the cuisine food in this scale. Zhang et al. (2024), investigated on wheat classification with 5 classes. They used near infrared (NIR) spectrometer to capture Two-image-sizeable correlation spectrum and analysis this data with EffucienNetB0, machine learning algorithms and their models. Deep learning model that they develop could classification five class of wheat with 100% accuracy. Razavi et al. (2024), worked on recognition and classification system based on deep learning and transfer learning algorithms for rice cultivars. Rice quality was important for human life so they combined deep learning and transfer learning for classification 6 classes of rice. But in this investigation, The data set creating challenges in recognition system, on one hand decreasing computing and training time, induced to design and developed the best architecture between 6 popular deep learning architectures. After balancing data set and complete data augmentation and preprocessing technique. Hyperparameter of model should be improved and efforts for designing best fully connected layer was started. Completing all the task allowed to propose new improved deep learning architecture for specific data set of food. Developed ResNet50 could recognition quality and classification rice with 98.13% accuracy. Recognizing food and consumed food was difficult because of, big data set, incomplete food, multi object, multi situations and All investigations have been done based on complete object and did not present improved model for recognize food in multi volume, so ResNet-50S was made. ResNet-50S was constructed from adjusted hyperparameter and specific fully connected layer. This model could recognize 16 class of foods with 97.25% et al.accuracy and 0.2% loss at 5 epochs.

## 6 Conclusion

Food authenticity, quality, and safety issues such as fraud, adulteration, poisoning, and food terrorism have created new tasks and challenges for food recognition and classification science. As consumers become increasingly concerned about the correct information and quality of food, the food industry is prompted to implement more effective measures to ensure food information. Food consumption significantly affects health, energy balance, environmental sustainability, and economic sectors. Proper management of food production and consumption is essential to mitigate issues like environmental degradation and public health challenges. An intelligent system has been developed to track food intake, enhance awareness, and reduce disorder and obesity by controlling consumption patterns. This system fosters individual responsibility while contributing to collective efforts toward sustainability and public health improvement. This system leverages machine vision and deep learning technologies to analyze food and food consumption from initiation to completion. Deep learning, with its diverse architectures, offers powerful tools for analyzing complex datasets. In this study, various deep learning architectures were employed to examine a specific dataset collected from consumed food and food items. The performance of these architectures, including ResNet50S, is detailed in Fig. 12. The analysis was conducted in two phases: first, a dataset comprising 16 food classes with a total of 12,000 images, derived from video analysis of food consumption, was analyzed using the ResNet50 algorithm. The initial model achieved an accuracy of 70%. After optimizing the hyperparameters and adjusting the fully connected layer, the accuracy improved significantly to 97.25%, with precision, recall, and F1-score reaching 96.3%, 97%, and 97%, respectively. The results demonstrate the potential of intelligent systems for accurately identifying consumed food items through the integration of machine vision and deep learning. These findings underscore the system's capability to enhance food monitoring and control processes, thereby contributing to more efficient and sustainable food consumption management.

## CRediT authorship contribution statement

**Pouya Bohlol:** Conceptualization, Investigation, Validation, Writing – original draft. **Soleiman Hosseinpour:** Supervision, Writing – review & editing. **Mahmoud Soltani Firouz:** Writing – review & editing.

## Declaration of competing interest

The authors declare that they have no known competing financial interests or personal relationships that could have appeared to influence the work reported in this paper.

## Data Availability

I shared the data with google derive link.
